# Diffuse Large B-Cell Lymphoma in the Older and Frail Patient

**DOI:** 10.3390/cancers17050885

**Published:** 2025-03-05

**Authors:** Emily C. Ayers, Sonali M. Smith

**Affiliations:** 1 Division of Hematology and Oncology, University of Virginia School of Medicine, Charlottesville, VA 22903, USA; eca2t@uvahealth.org; 2 Section of Hematology/Oncology, Department of Medicine, University of Chicago, Chicago, IL 60637, USA

**Keywords:** diffuse large B-cell lymphoma, frailty assessment, geriatric assessment, older adults

## Abstract

Outcomes in older, unfit, and frail patients with diffuse large B-cell lymphoma (DLBCL) are inferior compared to younger and fit patients. Despite tremendous progress in the treatment landscape of DLBCL, few clinical trials have focused specifically on this high-risk patient population. This review focuses on the pathophysiology unique to the older patient with DLBCL, as well as the evidence behind current treatment approaches. This article also aims to highlight emerging prognostic tools and novel treatment strategies that may improve the outcomes in this patient cohort in the future.

## Simple Summary

Diffuse large B-cell lymphoma is the most commonly diagnosed non-Hodgkin lymphoma and often affects older patients. Despite the higher prevalence of this disease in older patients or those with comorbidities, there is no established standard of care for patients who cannot tolerate the traditional chemotherapy regimens used in younger or more fit patients. Consequently, clinical outcomes are unfortunately inferior. Furthermore, the inclusion of elderly or frail patients in clinical trials has significantly lagged behind that of younger patient subgroups. This article reviews the existing evidence supporting the different treatment approaches for older or frail patients with diffuse large B-cell lymphoma. We highlight novel treatment approaches, either recently approved or under investigation showing promising efficacy and safety.

## 1. Introduction

Diffuse large B-cell lymphoma is the most common non-Hodgkin lymphoma, with approximately 20,000 new cases diagnosed annually in the United States. There has been great progress in both diagnostic and therapeutic approaches, and the majority of patients can expect to be effectively cured with modern chemoimmunotherapy. Unfortunately, these advancements have not extended equally to older or more frail patients. Whereas younger patients have experienced a 28% improvement in survival, patients over the age of 75 have seen only a 13% improvement in the modern era compared to the 1990s [1]. The five-year overall survival (OS) rate in patients over the age of 65 is 54.3%, compared to 78.4% in younger patients [2]. Outcomes are even more dismal for patients over the age of 80, where up to one-third of patients never receive treatment despite the possibility for cure [2].

The challenges of managing DLBCL in older patients are related to frequent comorbidities, social factors impacting access, limited representation in clinical trials, and, perhaps most significantly, the inability to align patient frailty with an optimal treatment approach in an individualized manner. The lack of prospective data is an important limitation. Fewer than 5% of studies in hematologic malignancies are specifically developed for older patients. Furthermore, simply including older patients in trials designed for a younger population has proved problematic when interpreting results, as there is increased toxicity, treatment discontinuations, and disparate outcomes between age groups. In this study, we will focus on the current therapeutic approaches and their challenges in this setting, the biology of DLBCL in this subgroup, and ongoing efforts to optimize treatment for older patients. A comprehensive search of the existing literature was conducted, with prioritization of prospective therapeutic trials in older adults with DLBCL, retrospective analyses dedicated to older adults with DLBCL, papers defining frailty assessment in DLBCL, and other publications regarding treatment of older adults with cancer.

### 1.1. DLBCL Pathobiology in Older Adults

Although not fully elucidated, the biology of DLBCL in older adults appears distinct from that of younger patient populations. Several validated adverse prognostic factors increase with age. For instance, in one study using gene expression profiling in 131 DLBCL patients over the age of 50 years, the incidence of activated B-cell subtype (ABC) DLBCL increased by 13.7% with each decade after the age of 50 years and accounted for up to 70% of DLBCL cases in patients over the age of 80 years [3]. The authors hypothesized that this trend may be reflective of the gradual loss of diversity in the B-cell repertoire during aging.

Higher rates of *MYC* expression, cytogenetic complexity, and mutational burden have also been associated with increased age at diagnosis, with a higher prevalence of *p53* mutation noted among older adults (62% vs. 17% in one study) [4,5]. It has also been well documented that older age is associated with EBV-related DLBCL, likely due to immune senescence associated with aging. Latent EBV infections result in chronic antigenic activation and immunosuppression, both of which are important in neoplastic processes. In the context of chronic EBV infection, the immunosenescence of aging—including T-cell dysregulation, loss of immunoregulation, and reduced output of new T-cells—is likely accelerated and plays a role in the pathogenesis of EBV-positive large cell lymphoma in the elderly [6].

Additionally, genomic analyses of DLBCL have consistently demonstrated abnormalities in genes involved in chromatin and epigenetics. Alteration in genes coding for DNA methylation, microRNA processing, chromatin remodeling, and post-translational modification have been reported in up to 25% of DLBCL cases [7,8]. These hallmark changes of aging are more prevalent in DLBCL among older patients and are theoretically targetable with the addition of novel agents to standard immunochemotherapy, as is being done in the ongoing SWOG 1918 clinical trial.

### 1.2. Patient Factors in Older Adults with DLBCL

Patient factors among older adults increase susceptibility to treatment toxicity. Older patients have decreased hematopoietic reserve, resulting in more cytopenias, and have higher rates of neutropenic fever, often despite receiving lower doses of chemotherapy and growth factor support. Up to 40% of older adults will have febrile neutropenia (FN) while receiving treatment with rituximab, cyclophosphamide, doxorubicin, vincristine, and prednisone (R-CHOP), and FN has been correlated with subsequently decreased dose intensity and worse outcomes [9]. Higher rates of peripheral neuropathy, hospitalizations, and cardiac dysfunction have also been observed in older patients receiving R-CHOP [10]. One report documented that over 20% of deaths occurring during therapy are toxicity-related in older adults [11].

Higher rates of pre-existing baseline comorbidities also increase toxicity risk. At the time of diagnosis, up to two-thirds of adults with non-Hodgkin lymphomas have at least one comorbidity [12]. An analysis has demonstrated that having multiple comorbidities is associated with decreased overall survival in DLBCL patients receiving treatment. Furthermore, curative chemoimmunotherapy is used less frequently in the presence of any comorbidity in favor of radiation alone in elderly patients with DLBCL. The increased treatment-related toxicity and resultant decreased dose intensity in this patient population has unfortunately resulted in disproportionately high mortality rates among older DLBCL patients receiving treatment. Data shows mortality rates of over 20% due to both treatment toxicity and disease progression [13,14].

### 1.3. Current Treatment Landscape: Initial Approach and Treatment

#### Frailty Assessment in DLBCL

**Figure 1:**
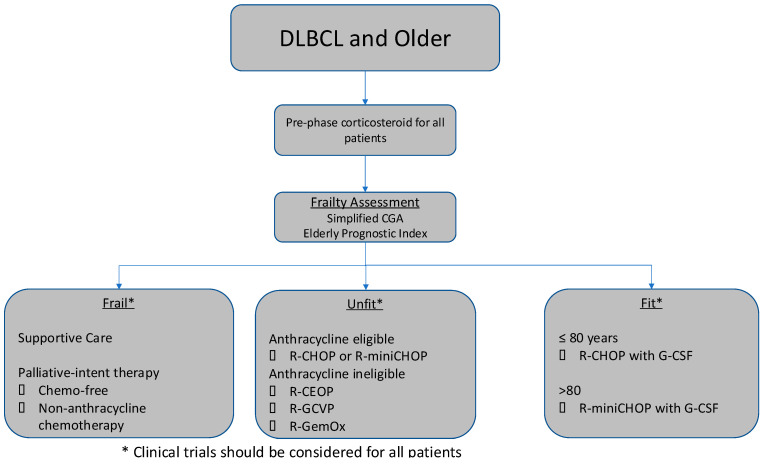
Current Treatment Algorithm in Older Patients with Previously Untreated DLBCL.

First, all treatment decisions need to be evaluated in the context of patient preferences and quality-of-life considerations, which are unique to each individual and cannot be ignored in this patient population. In recent years, there have been a number of efforts to optimize the treatment approach for older, unfit, or frail patients with DLBCL, which is summarized in Figure 1. Frailty metrics have been increasingly adopted in prospective clinical trials to minimize toxicity and maximize efficacy, although these tools are not yet widely used in standard-of-care practice. Frailty assessments provide an objective way to measure patient fitness, predict treatment tolerability and outcomes, and allow clinicians to tailor treatment decisions in order to maximize treatment efficacy and minimize toxicity in this patient population. Frailty assessments are useful tools that address multiple domains of potential comorbidities that might limit or exclude therapy with chemoimmunotherapy.

The Fondazione Italiana Linfomi (FIL) optimized the original geriatric assessment tool with a prospective multicenter observational study, the Elderly Project, which developed and validated the Elderly Prognostic Index (EPI) [15]. The EPI has been validated as a meaningful tool to assess both patient fitness and frailty and to prognosticate outcomes with first-line treatment in DLBCL [15]. The EPI relies on the simplified comprehensive geriatric assessment (Table 1) to classify patients as fit, unfit, or frail according to patient age, comorbidity score, and ability to complete activities of daily living. Patients are categorized into sCGA groups according to fitness and age. Group 1: Fit or < 80 years old and unfit, Group 2: unfit and ≥80 or frail and <80, and Group 3: frail and older than 80. The model then incorporates this assessment with clinical and biologic factors, including the IPI score and hemoglobin level, to create the EPI, which is highly predictive of overall survival. The EPI categorizes patients into three categories with distinct 3-year OS rates of 87%, 69%, and 42% for patients in the low, intermediate, and high-risk categories, respectively (Table 2). Going forward, real-time incorporation of the EPI may help individualize treatment intensity for older adults. S1918, an ongoing prospective randomized trial in older adults, is prospectively validating frailty assessments using an online FIL tool in development.

### 1.4. Treatment of the Fit and Unfit Older Adult

When treating an unfit or fit patient, the treating physician must first consider whether the patient is eligible for treatment with an anthracycline-containing regimen. Both fit and unfit patients should be assessed for anthracycline eligibility, as this remains the standard of care based on a number of retrospective studies. Inclusion of anthracyclines has demonstrated superior progression-free survival (PFS) and OS compared to palliative regimens without anthracyclines [16]. A large database study of more than 10,000 patients with DLBCL demonstrated improved 3-year OS of 63 vs. 44% with the inclusion of anthracycline [17]. Furthermore, while outcomes among unfit patients may be inferior to those of fit patients, unfit patients receiving anthracycline have improved outcomes compared to patients receiving palliative-intent treatments (1-year OS: of 66% vs. 19%) [18]. Multiple observational studies have described anthracycline avoidance in elderly patients, often without the use of formal geriatric or cardiac risk assessment, which likely leads to the undertreatment of select patients [19,20]. We emphasize that even among patients deemed “unfit” according to geriatric assessment, a specific cardiovascular risk assessment should be undertaken to definitively determine anthracycline eligibility. Whenever possible, consultation with cardio-oncology specialists should be pursued to optimize patient risk.

### 1.5. Prephase Treatment

The majority of mortality events occur within the first cycle of treatment in this patient population [21]. Prephase treatment with prednisone, with or without 1mg vincristine, has significantly reduced treatment and disease-related mortality and should be universally considered. Inclusion of 7 days of oral prednisone prior to cycle 1 of immunochemotherapy decreases therapy-related deaths and the incidence of tumor lysis syndrome, and shortens the duration of neutropenia. Additionally, pre-treatment with corticosteroids can improve the performance status of patients who may not have previously been candidates for curative-intent chemoimmunotherapy.

### 1.6. Does Dose Intensity Matter?

Among fit patients, the importance of dose intensity seems to be age-dependent. In patients over the age of 80 years, there is no consistent data that full-dose intensity is superior to a reduced-dose approach. A systematic review of over 4000 elderly patients with DLBCL found that dose intensity had a differential impact across different age groups [22]. Specifically, among patients ≥ 80 years old, there was no significant difference in PFS and OS according to dose intensity. Additional studies have demonstrated comparable outcomes in patients over the age of 80 years with dose intensity < 80%, using competing risk models to suggest that R-miniCHOP is a reasonable approach for patients ≥ 80 years old [23]. Thus, in the absence of any prospective randomized data, one reasonable approach in this age group is to start with dose-reduced R-CHOP, with the plan to increase incrementally depending upon the patient’s tolerability of each cycle.

In contrast, in fit patients younger than 80 years, more compelling and reproducible data emphasizes the importance of dose intensity. A number of studies have demonstrated inferior survival outcomes in patients 70–79 years old receiving reduced-intensity immunochemotherapy. One retrospective study of 690 elderly patients treated with curative intent demonstrated significantly inferior PFS and OS, as well as higher relapse rates, in patients younger than 80 years who received a dose intensity of <80% [23]. In patients younger than 80 with an intended dose intensity (IDI) of at least 80%, 70% of patients were alive without relapse, compared to only 50% of patients with an IDI of <80% (*p* = 0.004). In contrast, among patients older than 80 years, 57% and 49% were alive without relapse with an IDI of <80% and ≥80%, respectively (*p* = 0.04). Similarly, Tucci et al. demonstrated that the relapse rate increases significantly with a relative dose intensity of <70% in a prospective study of 173 elderly patients receiving either curative or palliative-intent treatment [24].

The standard of care for unfit patients is even less clear, as the benefit of curative-intent doses is uncertain. While one group did find that unfit patients can benefit from full-dose anthracycline, Merli et al. demonstrated that survival is similar within EPI groups, whether patients receive full-dose or reduced-dose intensity [15,24]. While it seems that the inclusion of anthracycline provides benefit, the importance and feasibility of dose intensity specifically in unfit patients is unknown.

### 1.7. Treatment Naïve DLBCL: Incorporation of Novel Agents Either With or Without Standard Anthracycline-Based Chemoimmunotherapy

The standard approach to patients ≥ 80 years old with DLBCL was arguably established by a small multinational prospective phase II trial of R-mini-CHOP [13], with the treatment regimen and dosing set as 375 mg/m^2^ rituximab, 400 mg/m^2^ cyclophosphamide, 25 mg/m^2^ doxorubicin, and 1 mg vincristine on day 1 of each cycle, and 40 mg/m^2^ prednisone on days 1–5. Among 149 treated patients with a median age of 83 years, the median overall survival was 29 months, and the 2-year overall survival was 59%. There were 58 deaths, with approximately half related to disease progression and 12 deemed treatment-related; of note, 13 patients (9%) died during the first cycle. Subsequent studies have supported routine incorporation of “pre-phase” steroids for older patients to improve performance status and overall function, albeit on a transient basis. Building on an R-mini-CHOP backbone is one approach to improving outcomes. Unfortunately, the multicenter, randomized phase III study of R-miniCHOP vs. R-miniCHOP plus lenalidomide by the Lymphoma Study Association was a negative trial [25]. This trial included 249 patients with a median age of 83 years and demonstrated no difference in OS (66% with R-miniCHOP vs. 65.7% with R-miniCHOP plus lenalidomide), regardless of cell of origin.

Currently, there are several ongoing prospective randomized trials with R-mini-CHOP as the backbone. SWOG 1918 is the first phase III randomized study conducted via the United States Intergroup mechanism in patients over the age of 75 years with DLBCL, randomizing patients to R-miniCHOP vs. R-miniCHOP plus oral azacitadine, an oral hypomethylating agent [26]. This study is novel in its prospective integration of the original FIL frailty tool, as well as serial comprehensive geriatric assessments. The POLAR BEAR study tests polatuzumab vedotin in the frontline treatment of this patient group by randomizing between Pola-R-miniCHP and R-miniCHOP [27]. Initial safety results suggest that the inclusion of polatuzumab vedotin is associated with higher rates of gastrointestinal adverse events (31% vs. 16%) but overall similar grade 3–4 hematologic toxicity. Peripheral neuropathy was similar between both arms, with 13% grade 1–2 neuropathy in the R-miniCHOP arm and 15% in the Pola-R-miniCHP arm. Efficacy data are not yet available for either trial.

“Chemo-free” regimens have been investigated in this setting, including CD20xCD3 bispecific antibodies and antibody-drug conjugates (ADC). Mosunetuzumab, a bispecific antibody, has been investigated both as monotherapy and in combination with polatuzumab vedotin, a CD79b-directed ADC, in frail patients with previously untreated DLBCL. As monotherapy, mosunetuzumab demonstrated a CR rate of 43% with a median duration of CR of 16 months [28]. With the combination regimen, mosunetuzumab plus polatuzumab resulted in a CR rate of 45% [29]. Loncastuximab-tesirine, a CD19-directed ADC, has also been evaluated in this patient cohort and demonstrated a CR rate of 59% in combination with rituximab in frail or unfit elderly patients in the LOTIS-9 study. Additional efforts are underway to investigate chemo-free options for these patients, including tafasitamab plus lenalidomide in patients over 80 years of age with previously untreated DLBCL(NCT04974216).

However, even with these chemo-free options, unexpected toxicities were uncovered in the unfit or frail patient population. For example, in the mosunetuzumab plus polatuzumab combination, fatal adverse events occurred in 14% of patients, including 7% mortality from COVID-19 infection [29]. Similarly, the LOTIS-9 study was terminated early due to unforeseen fatalities from respiratory infections.

### 1.8. Treatment of the Frail Older Adult

In frail patients, it is generally accepted that the morbidity associated with even reduced-dose immunochemotherapy is unacceptably high and that curative-intent therapy may not be feasible. As such, frail patients should be evaluated for palliative-intent treatments. There have been a number of efforts to evaluate chemotherapy-free regimens in this patient population.

For example, the FIL group investigated lenalidomide plus rituximab in frail older patients with previously untreated DLBCL in a phase II single-arm study [30]. The trial demonstrated a reasonable complete response rate of 28%, with a median PFS of 14 months. However, even with this chemo-free approach, grade 3–4 hematologic and nonhematologic toxicity was reported in 38% and 45%, respectively. Another chemo-free regimen includes ibrutinib in combination with lenalidomide and rituximab, which demonstrated a complete response rate of 56.7% with a 2-year PFS of 53.3% in a cohort of 30 patients. This group reported grade 3–4 hematologic toxicity of 23% and a rate of atrial fibrillation of 10%. Additional novel chemo-free approaches under active investigation are described below and summarized in Table 3.

### 1.9. Current Treatment Landscape: Treatment of Relapsed/Refractory Disease

At least one-third of patients with DLBCL will develop relapsed disease and require further treatment; this proportion is even higher in older adults. While both autologous stem cell transplant and CAR T-cell therapy are curative options for some patients, many older patients may not be eligible for these approaches due to comorbidities or lack of access. In general, autologous transplant may be considered in fit patients up to age 75 with chemosensitive disease, where transplant has shown a 2-year OS of 79% [34].

The revolutionary adoption of CAR T-cell therapy for second- and third-line (and beyond) treatment has expanded options for patients, although this treatment remains underexplored specifically in an older patient population. In the pivotal ZUMA-1 and TRANSFORM study of CAR T-cell therapy vs. standard-of-care salvage chemotherapy and autologous stem cell transplant, only 22% and 39% of patients in the investigational arms were over the age of 65 years, respectively [35,36]. Furthermore, there were no patients over the age of 75 included in the TRANSFORM study, making results hard to extrapolate to the patient population of interest here. Real-world evidence has shown shorter event-free survival in patients over the age of 75 receiving commercial CAR T-cell therapy, with a 12-month EFS of only 34%, compared to 43% and 52% in patients aged 65–69 and 70–74 years, respectively [37]. Other reports have demonstrated similar efficacy among patients ≥65 years old compared to those younger than 65, but data remain limited in patients over 70 years or those who are unfit [38].

Even in the relapsed setting, geriatric assessment has proven valuable in the prediction of patient outcomes. In patients receiving CAR T-cell therapy, cognitive/mobility impairment and comorbidities are associated with decreased overall survival and higher rates of cytokine release syndrome and neurotoxicity with treatment [39].

Palliative approaches with bispecific antibodies, antibody-drug conjugates, and targeted therapies—either alone or in combination with other novel agents—have promising potential in the relapsed/refractory space. However, the pivotal prospective studies with these agents were not tailored to older patients, and thus, we advise caution when extrapolating results from these trials.

### 1.10. Unique Challenges

#### Patients with Cardiac Dysfunction

In the absence of randomized data, there is no standard of care for patients with newly diagnosed DLBCL who are not candidates for anthracycline therapy. Substitution of doxorubicin with etoposide in R-CEOP has been proposed as a reasonable standard in this setting, with a similar 10-year PFS and disease-free survival compared to R-CHOP, although with a lower OS—likely reflective of underlying comorbidities in patients ineligible for anthracycline [40]. One smaller retrospective study suggests this regimen has more favorable outcomes among the germinal center vs. non-germinal center subtype, with a 2-year PFS of 32% vs. 80% [41].

Additionally, gemcitabine has been used as a substitution for anthracycline in R-GCVP, with acceptable outcomes [42]. In a multicenter phase II study of 62 patients with cardiac comorbidities and a median age of 76 years, the 2-year PFS and OS were 50% and 56%, respectively. A small case series reported promising activity with the use of lenalidomide in combination with cyclophosphamide, vincristine, and prednisone, with a CR rate of 80%; however, this series included only five patients treated in the first-line setting [43].

None of these regimens have been compared in a head-to-head manner. Interestingly, one study has shown that outcomes with non-anthracycline-containing regimens are comparable to outcomes in the pre-rituximab era [16]. In the coming years, it is likely we will see a shift away from cytotoxic chemotherapy in this patient population in favor of novel targeted and cellular therapies, which are likely more effective and potentially safer for these patients.

### 1.11. High-Grade B-Cell Lymphoma with Dual MYC and BCL2 Rearrangements

The treatment of HGBL with *MYC* and *BCL2* rearrangements in the elderly remains an unmet and unexplored need. Although not reviewed here, the standard for younger, fit patients with this entity remains intensified regimens such as dose-adjusted R-EPOCH [44,45]. Intensified regimens confer even higher risk for cytopenias and organ dysfunction; thus, for the reasons outlined earlier, these treatments carry an unacceptable toxicity in older adults, particularly if there is frailty at baseline. Although R-CHOP appears inferior when compared to more intensive immunochemotherapy for HGBL overall, there is limited data regarding the efficacy of R-miniCHOP or anthracycline-free regimens specifically among double-hit lymphoma patients. One retrospective study demonstrated a 24-month EFS of 33% and 40% with R-EPOCH and R-CHOP, respectively, in elderly patients with double-hit lymphoma [46]. These dismal outcomes highlight the need for additional therapeutic approaches, potentially including the use of cellular or T-cell engaging therapies in this high-risk patient population, if available and if patients are eligible.

## 2. Conclusions and Future Directions

Treatment of older, unfit, or frail patients with newly diagnosed DLBCL remains a therapeutic challenge. Maintaining curative intent in the context of advanced age and comorbidities is difficult with current anthracycline-based approaches. Despite exciting developments in DLBCL over the last few decades, improvements in outcomes among older adults have lagged behind. The difficulties in this setting stem both from patient-specific factors, which result in increased toxicity and decreased tolerability of treatments, as well as disease-specific factors, such as the enrichment of high-risk molecular and biologic factors within this group. These struggles are further compounded by the historical lack of data in this space due to the scarcity of clinical trials dedicated to older adults. However, as Table 3 shows, an increasing number of trials focused on older adults with DLBCL are now underway.

We hope that ongoing randomized studies in this space, including the S1918 and POLAR BEAR studies, will inform and improve future treatments for elderly DLBCL patients. It must be emphasized that geriatric assessments should be incorporated into clinical trials to improve individualized treatment delivery that optimizes the risk of toxicity with the benefit of lymphoma control. We also stress the importance of implementing frailty tools in standard-of-care practice to improve individualized treatment decisions and minimize treatment-related morbidity. The uptake of these tools has likely been somewhat curtailed by the assumption that geriatric assessment is cumbersome and time-intense. However, as we demonstrate here, the sCGA and EPI have vital roles in treating LBCL in older patients. Whenever possible, the incorporation of additional subspecialty care, such as palliative care colleagues or physical and occupational therapy specialists, may also be useful. As the treatment landscape continues to shift in favor of more novel and biologically rational approaches with diminished toxicity, we hope to see advances in the care of older patients with DLBCL.

## Figures and Tables

**Table 1:** Simplified Comprehensive Geriatric Assessment14.

Factor	Fit	Unfit	Frail
ADL	6	5	≤4
IADL	8	6–7	≤5
CIRS-G	0 of score ≥ 3 ≤5 of score 2	0 of score ≥ 3 5–8 of score 2	≥1 of score ≥ 3 >8 of score 2
Age		≥80 and Fit	≥80 and Unfit

ADL: activities of daily living; IADL: instrumental activities of daily living; CIRS-G: cumulative illness rating scale-geriatric.

**Table 2:** Elderly Prognostic Index [15].

Factor	Points	EPI Score	3-Year OS
sCGA Group 1	0	Low risk: 0–1	87%
sCGA Group 2	2		
sCGA Group 3	3	Intermediate risk: 2–4	69%
IPI 0–1	0		
IPI 2	1	High risk: 5–7	42%
IPI 3–5	3		
Hgb < 12 g/dL	1		

IPI: international prognostic index; Hgb: hemoglobin.

**Table 3:** Completed and Ongoing Prospective Studies of Elderly Patients with DLBCL.

Trial Number	Phase	Patient Population	Intervention	N	Median Age	Outcome
NCT01990144 [31]	II	>70 yo, frail by CGA	Bendamustine 90 mg/m^2^ + rituximab	45	81	CR 53%, ORR 62%, mPFS 10mo
NCT02955823 [30]	II	>69 yo, frail by sCGA	Lenalidomide 20 mg + rituximab	68	83	CR 28%, ORR 51%, mPFS 14mo
NCT01195714 [14]	II	>80 yo	Ofatumumab + miniCHOP	120	83	CR 56%, ORR 68%, 2-yr OS 65%, mOS not reached
NCT03943901 [32]	II	≥75 yo or 70–74 with elevated CIRS	Split dose R-CHOP	14	81	CR 71%
NCT03949062 [33]	II	≥75 yo and unfit or frail	Ibrutinib + lenalidomide + rituximab	30	80	CR 56.7%, ORR 66.7%, 20year PFS 53.3%
NCT03677154 [29]	I/II	≥80 yo or 65–79 yo and unfit	Mosunetuzumab + polatuzumab	108	81	CR 45%, ORR 55%
NCT03677154 [28]	I/II	≥80 yo or 65–79 yo and unfit	Mosunetuzumab	54	83	CR 35%, ORR 43%, 12-month PFS 39%
Ongoing Studies						
NCT04799275 [26]	II/III	≥75yo	R-miniCHOP +/− oral azacitadine	Planned 422		
NCT04332822	III	>80 yo or 75–80 yo and frail by sCGA	R-miniCHOP vs. R-pola-miniCHOP			
NCT0616729	II	≥70 yo and unfit or frail by CGA	Polatuzumab+ lenalidomide/rituximab			
NCT05144009	II	Unfit and frail	Loncastuximab + rituximab	40		

CGA: Comprehensive geriatric assessment; sCGA: simplified comprehensive geriatric assessment; CIRS: cumulative illness rating scale.

## Data Availability

Not applicable.
